# Formulation Optimization, Multi-Component Compounding Mechanisms, and Regeneration Insights of a Waste Vegetable Oil-Based Bitumen Regenerant

**DOI:** 10.3390/ma19112323

**Published:** 2026-05-31

**Authors:** Tianhao Zhao, Zhengqi Zhang, Chang Lu, Wei Lu, Zhixin Liu, Songxiang Zhu

**Affiliations:** 1 Highway College, Chang’an University, Xi’an 710064, China; tian-hao-z@chd.edu.cn (T.Z.);; 2 School of Civil Engineering, Chongqing Jiaotong University, Chongqing 400074, China

**Keywords:** waste vegetable oil, formulation optimization, response surface methodology, IGMH analysis, energy decomposition, electronic structural characteristics

## Abstract

**Highlights:**

- A formulation optimization framework integrating multi-temperature rheological properties and interfacial water stability provides greater engineering applicability than traditional methods.
- The waste vegetable oil-based regenerant establishes a synergistic interaction network dominated by dispersion forces, supplemented by localized dispersion-driven stacking and hydrogen-bonding interactions.
- Molecular-level regeneration of aged asphaltenes involves aggregate depolymerization, enhancement of reversible deformation capacity, and reshaping of electronic structural characteristics.

**Abstract:**

Waste vegetable oil-based regenerants (WVO-Rs) are essential for sustainable asphalt pavements; however, their formulation optimization frameworks remain insufficient, and both the component synergy and the multi-component regeneration mechanism remain unclear. In this study, Response Surface Methodology was employed to optimize the WVO-R formulation by jointly considering the multi-temperature performance and interfacial water stability of the regenerated bitumen. Multi-scale performance tests and quantum chemical calculations were conducted to comprehensively evaluate its regeneration effectiveness and thermal behavior and to elucidate the underlying molecular mechanisms. The results indicate that the formulation optimization framework dominated by multi-temperature rheological properties and interfacial water stability exhibits superior engineering applicability compared with traditional methods, and the optimal WVO-R formulation corresponds to a mass ratio of WVO:DBP:CPR:SCA:ATO = 100:23.6:14.4:1.7:1. The WVO-R achieves optimal comprehensive regeneration at a dosage of 6–8%, exhibiting excellent thermal and storage stability along with uniform mixing. At the molecular level, the WVO-R forms a dynamic and stable molecular aggregate structure by integrating inherently stable components, leveraging the bipolar silane coupling agent to regulate critical polarity mismatches of dibutyl phthalate (DBP), and establishing a synergistic interaction network dominated by dispersion forces, supplemented by localized stacking and hydrogen-bonding interactions. On this basis, Oleic acid further depolymerizes aged asphaltene (AAS) aggregates through hydrogen bonding interactions, DBP enhances the reversible deformation capacity of AAS via π–π stacking effects, and the overall WVO-R components reshape the electronic structural characteristics of AAS to levels comparable to virgin asphaltene by smoothing the surface electrostatic potential gradient and suppressing electronic reactivity. Overall, this study establishes a systematic framework for WVO-Rs that integrates formulation optimization, regeneration performance evaluation, thermal behavior analysis, and molecular-level mechanism elucidation, thereby providing solid theoretical support for the efficient design and engineering application of bio-based bitumen regenerants.

## 1. Introduction

Environmental sustainability has become a central concern in the transportation sector, requiring pavement infrastructure to deliver high performance while minimizing carbon emissions and environmental impacts [1,2]. As a widely used paving material, bitumen involves substantial energy consumption and pollutant emissions during production and mixing [3,4], making the recycling of aged bitumen an effective strategy for sustainable road construction [5]. Nevertheless, long-term service causes severe stiffening and embrittlement of bitumen, markedly limiting its direct reuse in pavement applications [6]. Therefore, the application of bitumen regenerants is essential for restoring the fundamental properties of aged bitumen and enabling its effective reuse.

Traditional bitumen regenerants mainly consist of petroleum-based light oils, aromatic oils, and mineral oil materials [7]. However, these regenerants generally face limitations such as non-renewability, high energy consumption, and adverse environmental impacts. In contrast, bio-based regenerants, typically derived from vegetable oils, animal fats, plant oils, or their derivatives, offer advantages including high renewability and a low carbon footprint [8]. Among them, waste vegetable oil (WVO) is a representative bio-based regenerant, rich in light components such as fatty acids and esters [9]. It not only replenishes light fractions lost during bitumen aging but also promotes deagglomeration and dispersion of aged asphaltene aggregates [10,11]. Recently, WVO has emerged as a key focus of research in bitumen regeneration. For example, Zhilong Cao [12] utilized the synergistic effect of bio oil and isocyanate to restore the low-temperature and high-temperature properties of aged SBS bitumen, achieving dual regeneration of molecular structure and road performance. Hallizza Asli [13] experimentally confirmed that waste edible oil can serve as an effective bitumen regenerant, with its optimal dosage requiring adjustment based on the extent of bitumen aging. Tianyuan Yang [14] found that adding 10 wt% bio-oil optimally enhances the ductility of aged bitumen and restores its thermal expansion coefficient, whereas excessive addition decreases both thermal stability and ductility. Junfeng Su [15] successfully developed microcapsules for waste cooking oil using methanol–melamine–formaldehyde as the shell, exhibiting excellent thermal stability and structural durability, which enables in situ regeneration of aged bitumen. Jizhe Zhang [16] reported that vegetable oil, particularly in the form of a composite regenerant, effectively restored the rheological and viscoelastic properties of aged binders and regenerated asphalt mixtures containing 60% RAP; however, the dynamic modulus and fatigue life did not fully recover to the levels of unaged mixtures. Ran Zhang [17] showed that bio-oil derived from waste wood, which is rich in light components, substantially restored the rutting resistance of aged bitumen, although not to its original level, and also improved its resistance to low-temperature cracking. However, when the bio-oil content exceeded 15%, its additional benefits to rheological properties became limited. Wenjun Bai [18] found that bio-bitumen derived from corn stover, castor oil, and soybean-based bio-oils enhanced low-temperature cracking resistance, although low-temperature fatigue performance was generally reduced. Yiyang Xue [19] demonstrated that waste vegetable oil (WVO) reduced bitumen viscosity and significantly improved its high-temperature performance, elasticity, and low-temperature properties under low-temperature, short-time mixing conditions.

Although WVO is widely recognized as a promising bitumen regenerant, its direct application still suffers from several notable limitations. First, its strong softening and diluting effects markedly reduce the high-temperature resistance to permanent deformation and the moisture resistance of regenerated bitumen [20,21]. Second, WVO alone is insufficient to compensate for the loss of plastic deformation capacity resulting from asphaltene condensation in aged bitumen [22]. Moreover, it is prone to volatilization and secondary aging during service [23]. To address these drawbacks, recent studies have focused on compounding WVO with functional additives, such as plasticizers, thickeners, and antioxidants. For instance, Zhang [24] combined WVO with plasticizers and antioxidants, demonstrating that the composite regenerant not only restored the fundamental properties of aged bitumen but also significantly improved its fatigue resistance and low-temperature cracking resistance. Bo Li [25] developed a regenerant composed of WVO, plasticizers, toughening agents, and petroleum resin, which effectively diluted the carbonyl and sulfoxide functional groups in UV-aged bitumen. Ying Fang [11] formulated a compound regenerant by blending waste soybean oil with permeation, polymer, and functional components, achieving superior regeneration performance compared with commercial regenerants. In parallel, increasing attention has been devoted to elucidating the regeneration mechanisms of WVO-based regenerants (WVO-Rs). Dongliang Hu [10] used palmitic acid as a model bio-based regenerant and employed classical molecular simulations to investigate the dissolution behavior of asphalt molecules in n-heptane, confirming the restorative role of the regenerant. Ying Fang [26] reported that fatty acid glycerides most effectively depolymerize asphaltene aggregates owing to their low polarity and weak electrostatic interactions, which disrupt intermolecular associations. Mei Deng [27] represented bio-based regenerants using oleic acid, glycerol, and palmitic acid and applied density functional theory (DFT), demonstrating that dispersion interactions and hydrogen bonding are the dominant driving forces for the depolymerization of aged asphaltene aggregates. However, most studies rely solely on basic bitumen indices to determine the optimal formulation, which is insufficient for comprehensively assessing regeneration effectiveness across different formulations. More importantly, the interactions between WVO and other functional components have not been systematically investigated, leading to a limited understanding of the multi-component compounding mechanisms of WVO-Rs. Furthermore, the multi-component regeneration mechanisms of the formulated WVO-Rs have yet to be fully elucidated.

Quantum chemical calculations offer distinct advantages in elucidating molecular-scale mechanisms, as they enable quantitative characterization of interaction energies and their underlying nature, while also providing intuitive visualization of the spatial features of weak intermolecular interactions [28]. Farideh Pahlavan [29] employed quantum chemical calculations to demonstrate that the primary components of bio-based regenerants can mitigate the polar substitution effects induced during oxidation. Guannan Li [30] used quantum chemical methods to calculate the dipole moments, electrostatic potentials, and HOMO-LUMO energy gaps of 50 asphalt molecular models, revealing that donor atoms located in benzene-rich regions are more susceptible to electrophilic attack. Hu [10] combined density functional theory (DFT) with wavefunction analysis to show that bio-based regenerants weaken hydrogen bonding, thereby reducing electrostatic interactions and alleviating molecular aggregation. Deng [22] integrated DFT and FTIR analyses to demonstrate that bio-based regenerants disrupt asphaltene aggregation through synergistic hydrogen bonding and dispersion interactions, exhibiting a stronger depolymerization effect than petroleum-based regenerants.

Based on the identified research gaps and the proposed methodology, the preliminary regeneration dosage of WVO was first determined according to the basic physical properties of the regenerated bitumen. Subsequently, Response Surface Methodology (RSM) was further employed to optimize the functional composition ratio of the WVO-R, with the high-, medium-, and low-temperature performance of the regenerated bitumen, as well as its interfacial water stability with aggregates, serving as the response variables. Next, multi-scale performance tests were conducted to comprehensively evaluate the regeneration efficiency, optimal dosage, and thermal behavior of the WVO-R with the selected formulation. In quantum chemical simulations, molecular models of the WVO-R components and asphaltenes were constructed, and the global minimum points of their molecular complexes were identified using *ABCluster 3.3*. *Gaussian 16* was then employed to perform geometric optimization, vibrational frequency analysis, and single-point energy calculations, while *Multiwfn 3.8* was used for wavefunction analysis of the optimized molecular complexes. This approach enabled the identification of the primary intermolecular interaction modes and electronic structural characteristics, providing molecular-level insights into the multi-component compounding and regeneration mechanisms of the WVO-R. Overall, this study established a systematic research framework for WVO-Rs that integrates formulation optimization, regeneration performance evaluation, thermal behavior analysis, and molecular-level mechanism elucidation. 

## 2. Objective and Scope

The specific research objectives and scope of this study are as follows:

(1) Optimize the multi-component formulation of the WVO-R using RSM, with G^*^/sinδ, N_f_, m/S, ER as key response indicators;
(2) Compare the regeneration effectiveness of the optimal WVO-R formulations obtained using the traditional optimization approach and the optimization framework developed in this study;
(3) Comprehensively evaluate the regeneration efficiency, optimal dosage range, and thermal behavior of the optimized WVO-R through multi-scale performance tests;
(4) Investigate the electronic structure characteristics of the WVO-R component molecules and their complexes with aged asphaltenes, focusing on molecular polarity and HOMO-LUMO energy gap variations;
(5) Analyze the interaction characteristics among the WVO-R components and between them with virgin/aged asphaltenes, focusing on noncovalent interactions such as dispersion force, hydrogen bond, and dispersion-driven stacking;
(6) Discuss the multi-component compounding and AAS regeneration mechanisms of the WVO-R at the molecular level by integrating systematic analyses of electronic structure characteristics and intermolecular interactions.

## 3. Materials

### 3.1. Waste Vegetable Oil

**Figure 1:**
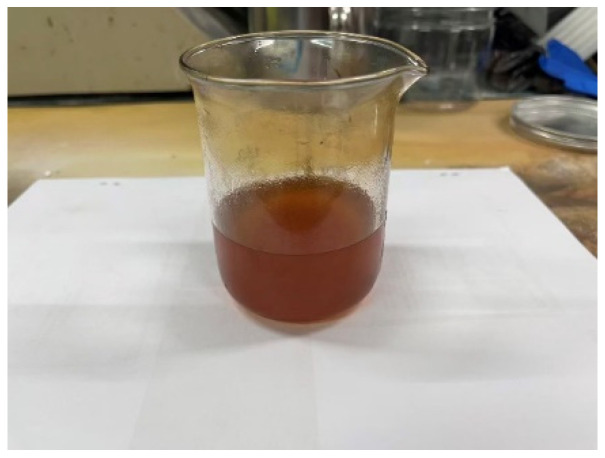
Appearance of WVO.

The waste vegetable oil (WVO) used in this study was soybean oil recovered from the catering industry and was pretreated by filtration, precipitation, and dehydration. Its appearance is brown, as shown in Figure 1.

Gas chromatography-mass spectrometry (GC-MS) [31,32] is used to identify the components of an unknown substance by separating its individual constituents and matching their mass-spectral fragments, and to quantify the content of each component based on the chromatographic peak area. In this study, the collected WVO was analyzed using a GC-MS system. The compositional analysis results of the WVO are presented in Table 1. It is evident that the unsaturated fatty acid (oleic acid) is the predominant component of the WVO, accounting for nearly 40% of its total content.

### 3.2. Functional Components of WVO-R

The functional components in WVO-R were selected based on a clear structure–function rationale. Specifically, dibutyl phthalate (DBP) molecules contain aromatic rings and flexible ester side chains, which can restore the plasticity of the rejuvenated system by molecular lubrication and increasing the free volume [33]. C5 petroleum resin (CPR) exhibits hydrophobicity and tack-enhancing characteristics [34,35], enabling it to improve the cohesion and adhesive strength of the regenerated system. Antioxidant 1076 (ATO) is a hindered phenolic antioxidant that scavenges free radicals and inhibits oxidation chain reactions [36], thereby retarding the further aging of WVO and regenerated bitumen. In addition, the silane coupling agent (SCA) enhances the interfacial stability of the WVO-R blended system through its bifunctional molecular structure [37]. Overall, the incorporation of these functional components synergistically compensates for the limitations associated with the use of WVO alone.

**Figure 2:**
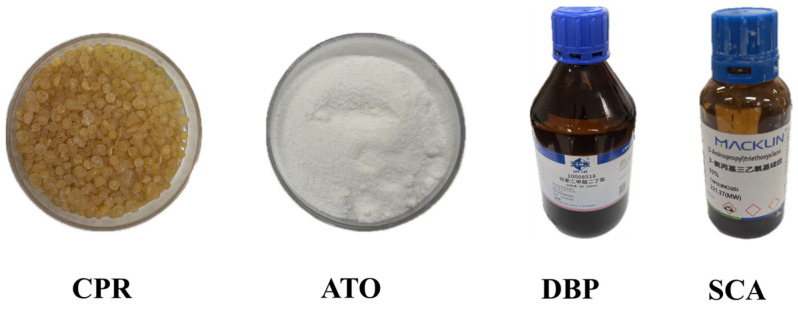
Appearance of functional components for WVO-R.

Accordingly, DBP, CPR, ATO, and SCA were used as the plasticizing, tackifying, antioxidant, and stabilizing components of WVO-R, respectively. The appearances of these functional components are shown in Figure 2. Specifically, CPR appears as light-yellow colloidal particles, ATO as a white powder, and both DBP and SCA as transparent liquids. Their basic technical properties are summarized in Table 2.

### 3.3. Long-Term Aged Bitumen

SK-70# bitumen was selected as the base material. Following the literature procedure [38,39] for simulating long-term bitumen aging, the unaged bitumen was first subjected to short-term aging via the rolling thin-film oven test (RTFOT), followed by long-term aging in a pressure aging vessel (PAV). The basic technical indicators of the unaged and long-term aged bitumen are listed in Table 3. It should be noted that all references to aged bitumen hereinafter refer to long-term aged bitumen.

## 4. Method

### 4.1. Preparation of Regenerated Bitumen and Waste Vegetable Oil-Based Regenerants

**Figure 3:**
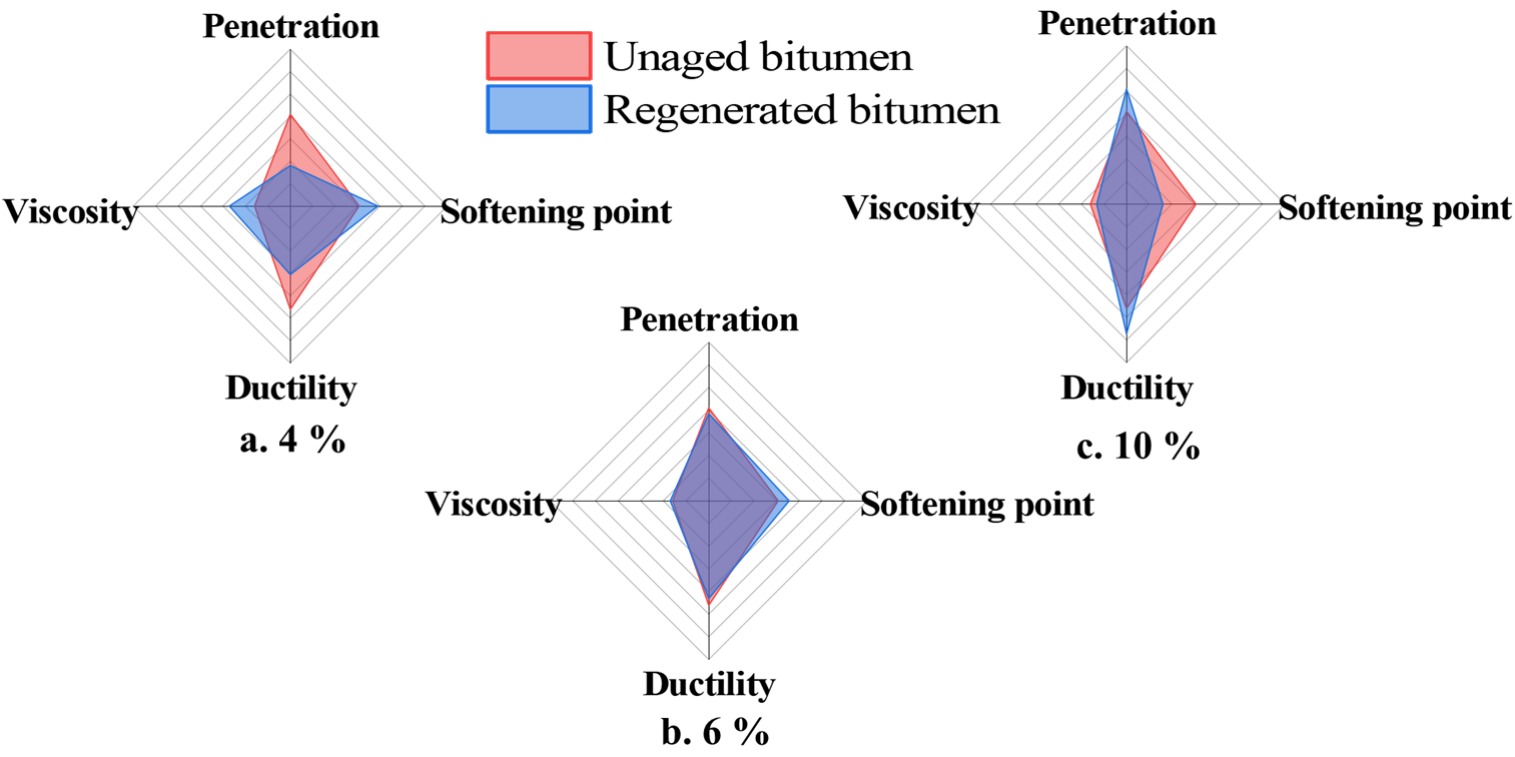
Basic properties of regenerated bitumen from WVO.

This study employed the conventional thermal regeneration method [40] to restore aged bitumen. The WVO was incorporated into the aged bitumen at mass ratios of 4%, 6%, and 10% to prepare regenerated bitumen, and their penetration, softening point, ductility, and viscosity were evaluated. The results show that a WVO content of 6% yields regenerated bitumen with basic properties most similar to those of the unaged bitumen, as illustrated in Figure 3. Consequently, the WVO-R regeneration dosage in the subsequent formulation design was preliminarily set at 6%.

**Figure 4:**
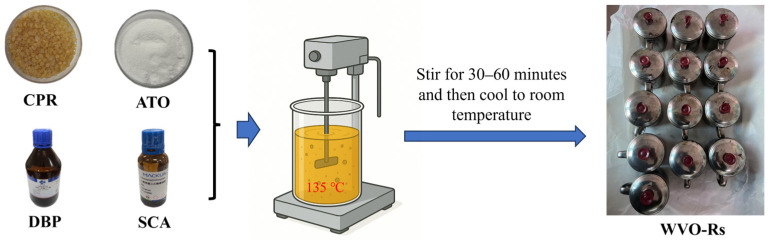
Production process of waste vegetable oil-based regenerants.

The preparation process of the WVO-Rs is illustrated in Figure 4. WVO (100 g) was heated to 135 °C, after which measured amounts of DBP, CPR, ATO, and SCA were added sequentially. The mixture was stirred for 30–60 min until homogeneous and then cooled to room temperature to obtain the regenerants.

### 4.2. Formulation Design of WVO-R Based on Response Surface Methodology

The dosage ranges of the WVO-R components were determined based on literature reports and their functional roles in the regeneration system. DBP and CPR were used as the major components, and their dosage ranges were set at 10–25 wt.% and 5–15 wt.%, respectively, according to Refs. [11,24,25], in which these levels exhibited effective regeneration performance. ATO was fixed at 1 wt.% because hindered phenolic antioxidants are generally effective at low dosages for scavenging free radicals and inhibiting oxidation chain reactions [41]. In this study, ATO served as an auxiliary antioxidant rather than a primary rheology-regulating component and was therefore kept constant to reduce the number of design variables. SCA was used as a low-dosage interfacial stabilizer rather than a bulk modifier. SCAs typically regulate interfacial interactions and improve compatibility and adhesion at relatively low dosages [42], its dosage range was limited to 1–3 wt.%.

Response surface methodology (RSM) [43] was employed in this study to optimize the proportions of the functional components in WVO-R. RSM was selected because it enables efficient, interpretable optimization of multiple variables and their interactions with a limited number of experiments [44]. The contents of DBP, CPR, and SCA were selected as influencing factors, while the high-temperature (G^*^/sinδ), medium-temperature (N_f_), low-temperature (m/S), and interfacial water stability (ER) indices of the regenerated bitumen were adopted as response variables. Detailed procedures for obtaining these response indicators are described in the following sections, and the specific experimental combinations along with the corresponding results are summarized in Table 4. It should be noted that the identical factor combinations shown in Table 4 represent intentionally replicated center-point runs in the RSM design, which were included to estimate pure experimental error, evaluate model adequacy, and improve the repeatability and statistical precision of the experimental results [45]. In addition, Design-Expert 13 was used for regression modeling and ANOVA of the four response indicators. The objective was to evaluate the significance, adequacy, and reliability of the developed models for prediction and optimization. Statistical significance was set at *p* < 0.05. Model performance was assessed using the model *p*-value, lack-of-fit, predicted R^2^, adjusted R^2^, and Adeq Precision.

### 4.3. Dynamic Shear Rheological Test and Bending Beam Rheological Test

**Figure 5:**
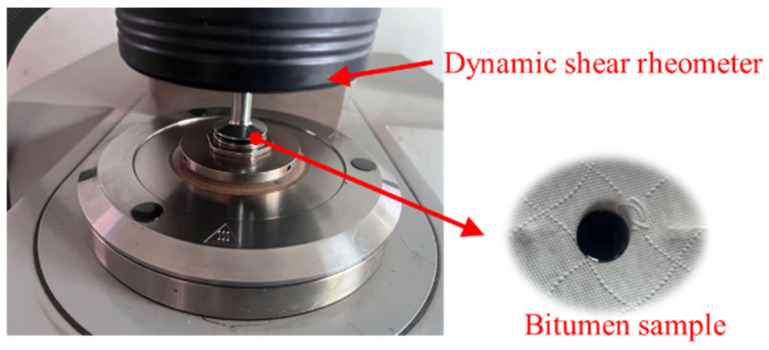
Experimental image of DSR.

**Figure 6:**
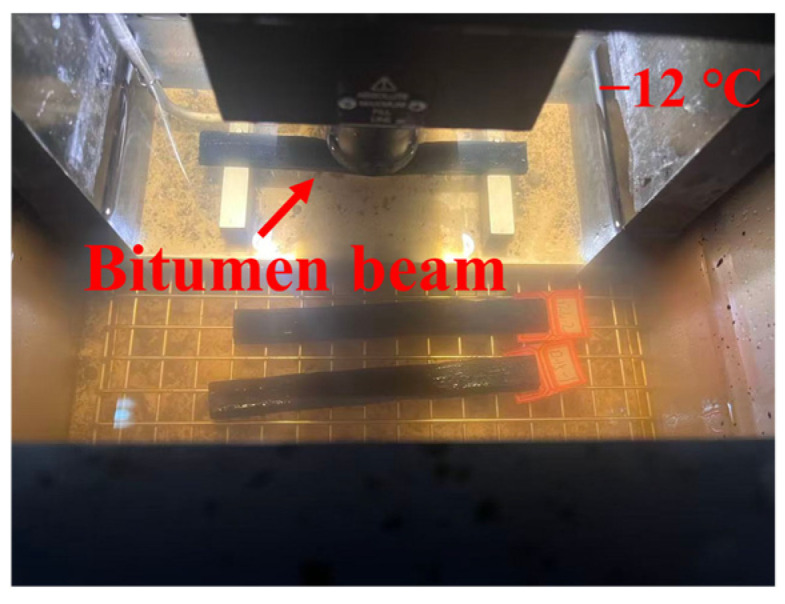
Experimental image of BBR.

This study employed a dynamic shear rheometer (DSR) and a bending beam rheometer (BBR) to assess the high-, medium-, and low-temperature performance of different regenerated bitumens, as shown in Figure 5 and Figure 6.

The high-temperature performance of regenerated bitumen was assessed using the temperature-scanning test of the DSR, with G^*^/sinδ at 60 °C serving as the evaluation index. G^*^/sinδ reflects the resistance to permanent deformation, with higher values indicating stronger rutting resistance [46]. Here, G^*^ represents the complex shear modulus, and sinδ denotes the sine of the phase angle. The test was conducted at a constant angular frequency of 10 rad/s.

The medium-temperature fatigue resistance of regenerated bitumen was evaluated using the linear amplitude sweep test of the DSR, with the predicted fatigue life (N_f_) [47] at a shear strain of 2.5% serving as the evaluation index. The test was conducted at a temperature of 28 °C and a frequency of 10 Hz. The calculation formula for N_f_ is shown in Equation (1).

(1) $$N_{f}=A\times {\left(\gamma_{max}\right)}^{B}$$

where A is the fatigue life coefficient, $\gamma_{max}$ is the maximum applied shear strain (%), B is the strain sensitivity index.

The low-temperature performance of regenerated bitumen was evaluated through the BBR test, with the comprehensive index m/S [48] at −12 °C employed to characterize its low-temperature crack resistance. Here, m represents the creep rate, and S denotes the creep stiffness (MPa). A higher m/S value indicates better low-temperature crack resistance.

### 4.4. Contact Angle Test

**Figure 7:**
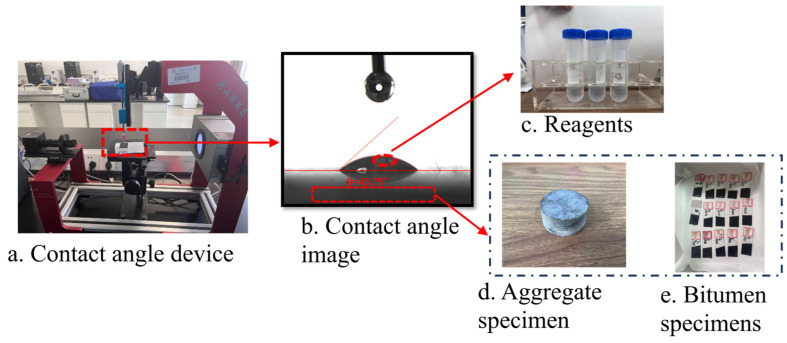
Contact angle test process.

The contact angle test was conducted at an ambient temperature of 20 °C using the sessile drop method [49]. Contact angles were measured using an SPCAXI-type contact angle goniometer, as shown in Figure 7a,b. Distilled water, formamide, and glycerol were used as probe liquids (Figure 7c). The aggregate specimen was composed of surface-polished basalt (arithmetic mean roughness = 1.4 μm), while the bitumen specimens were prepared using the hot-dip method [50], as illustrated in Figure 7d,e. Based on the measured contact angles, the surface energy parameters of the regenerated bitumen and basalt aggregate were calculated, and the Energy Ratio (ER)—a key indicator of water stability at the bitumen-aggregate interface—was subsequently determined using Equations (2)–(4).

(2) $$W_{\mathrm{as}}=\gamma_{a}+\gamma_{s}-\gamma_{as}=2\sqrt{\gamma_{a}^{d}\gamma_{s}^{d}}+2\sqrt{\gamma_{a}^{p}\gamma_{s}^{p}}$$

(3) $$W_{\mathrm{aws}}=W_{\mathrm{aw}}+W_{\mathrm{sw}}-W_{\mathrm{as}}=2\sqrt{\gamma_{a}^{d}\gamma_{w}^{d}}+2\sqrt{\gamma_{a}^{p}\gamma_{w}^{p}}+2\sqrt{\gamma_{s}^{d}\gamma_{w}^{d}}+2\sqrt{\gamma_{s}^{p}\gamma_{w}^{p}}-2\sqrt{\gamma_{a}^{d}\gamma_{s}^{d}}-2\sqrt{\gamma_{a}^{p}\gamma_{s}^{p}}$$

(4) $$\mathrm{ER}=\left|W_{\mathrm{as}}/W_{\mathrm{aws}}\right|$$

Here, $\gamma_{a}$ is the surface energy of bitumen, $\gamma_{s}$ denotes the surface energy of aggregate, $\gamma_{as}$ is the bitumen-aggregate interface energy, $W_{\mathrm{aw}}$ denotes the adhesion work of bitumen-water, $W_{\mathrm{sw}}$ indicates the adhesion work of aggregate-water, $W_{as}$ denotes the adhesion work of bitumen-aggregate, and $W_{aws}$ denotes the stripping work of bitumen-aggregate. In addition, $\gamma_{w}^{d}{,\gamma }_{w}^{p}$ indicates the dispersive and polar components of distilled water, $\gamma_{a}^{d}{,\gamma }_{a}^{p}$ denote the dispersive and polar components of bitumen, and $\gamma_{s}^{d},\gamma_{s}^{p}$ correspond to the dispersive and polar components of aggregate.

### 4.5. Differential Scanning Calorimetry Analysis

Differential scanning calorimetry (DSC) is a fundamental thermal analysis technique used to characterize the thermal stability and phase transition behavior of a material or system [51]. In this study, DSC analysis was conducted on both WVO and WVO-R to evaluate their thermal stability and homogeneity. The samples underwent a two-cycle “heating–cooling–reheating” scan under nitrogen protection to eliminate thermal history. The test temperature ranged from −80 °C to 150 °C, with heating and cooling rates of 10 °C/min.

## 5. Details of Quantum Chemical Calculations

### 5.1. Construction and Optimization of Molecular Models for WVO-R Components and Asphaltene

**Figure 8:**
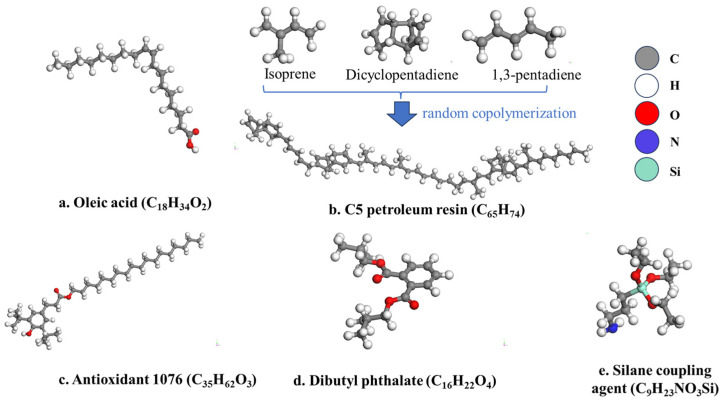
Molecular models for WVO-R components.

WVO readily undergoes hydrolysis under high-temperature and moisture-rich conditions, generating large amounts of free fatty acids [52]. GC-MS analysis in Section 3.1 also confirmed that oleic acid (OA) is the major component of the WVO. OA retains the key structural features of WVO-derived fatty acids, namely polar carboxyl groups and long hydrophobic alkyl chains. Therefore, OA was selected in this study as a simplified model compound for the WVO, and its molecular structure is shown in Figure 8a. C5 petroleum resin (CPR) is produced through the cationic polymerization of C5 olefins obtained from petroleum cracking, mainly including isoprene, cyclopentadiene, and 1,3-pentadiene [34], among which cyclopentadiene readily dimerizes into dicyclopentadiene [35]. Based on these characteristics, a random copolymer model (degree of polymerization = 10) was constructed for CPR using isoprene, dicyclopentadiene, and 1,3-pentadiene at a molar ratio of 4:3:3, as shown in Figure 8b. Representative molecular models of the antioxidant, plasticizer, and stabilizer are shown in Figure 8c–e.

**Figure 9:**
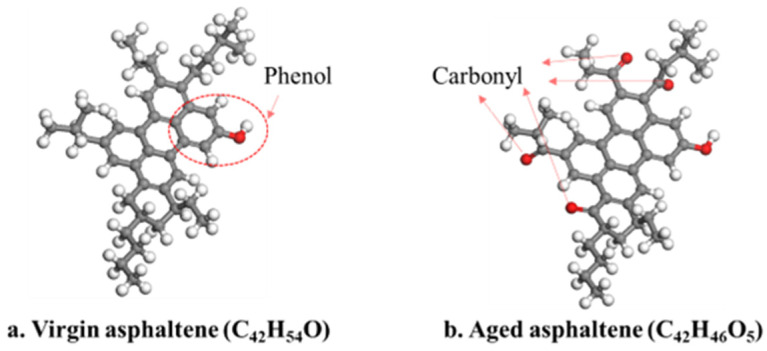
Molecular models for asphaltene.

Petroleum bitumen is a typical colloidal dispersion system, in which asphaltenes act as the core of the colloidal structure [53]. In this study, the phenolic asphaltene model (MW = 574) was selected as a representative structure because it incorporates the key features governing asphaltene behavior, including a condensed aromatic core, alkyl substituents, and polar heteroatom-containing functional groups, as illustrated in Figure 9a. Following the method reported in the literature [54], an aged asphaltene model was constructed by replacing tert-butyl hydrogen atoms with carbonyl (C=O) groups, as shown in Figure 9b.

All molecular models were subjected to frequency analysis and structural optimization using *Gaussian 16* at the B3LYP-D3/6-31G(d) level.

### 5.2. Construction and Optimization of Molecular Complex Models

The global minimum point corresponds to the most stable configuration of a molecular cluster, defining the optimal interaction mode and maximum intermolecular binding strength [55]. Identifying this global minimum point is a critical step for elucidating the dominant interaction mechanism and assessing the relative stability of molecular clusters [56]. *ABCluster 3.3* [57], an open-source program based on the artificial bee colony algorithm, is designed to locate the global minimum points of atomic and molecular clusters. In this study, the geom module of *ABCluster 3.3*, combined with the semi-empirical xTB method, was used to determine the optimal spatial arrangements and global minimum points of molecular complexes. The geom module enables global optimization for clusters containing both rigid and flexible units [58], while xTB rapidly predicts molecular geometries, energies, and related properties [59]. A total of 200 iterations were performed for each configuration search.

**Figure 10:**
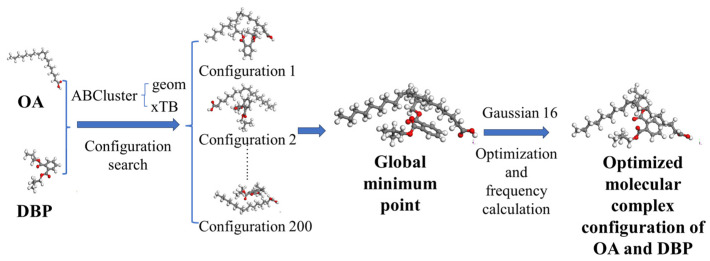
Construction and optimization process of molecular complex.

**Figure 11:**
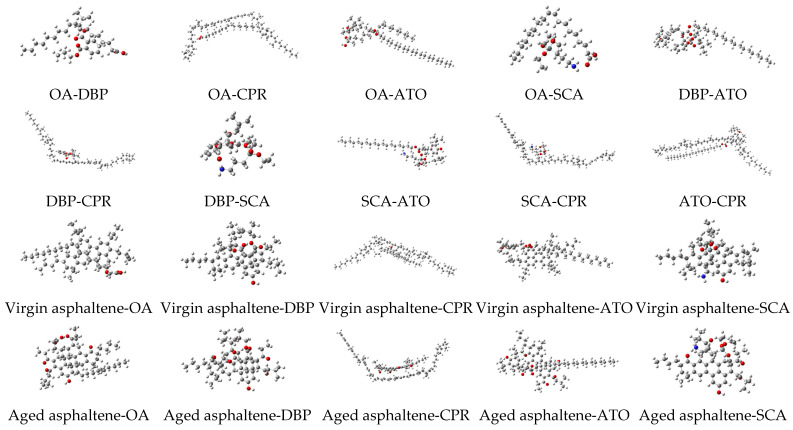
All optimized molecular complex configurations.

After identifying the global minimum point, the molecular complex was further optimized and subjected to vibrational frequency analysis using *Gaussian 16* at the B3LYP-D3/6-31G(d) level to confirm the absence of imaginary frequencies. As an example, the construction and optimization process of the OA-DBP molecular complex is illustrated in Figure 10. The optimized configurations of all molecular complexes considered in this study are presented in Figure 11.

### 5.3. Intermolecular Interaction Analysis Methods

Single-point energy calculations were performed on the optimized molecular complexes using a higher-level functional and basis set combination (M06-2X/def2-SVPP) in *Gaussian 16*. The resulting wavefunctions were subsequently analyzed using the *Multiwfn 3.8* program [60] to investigate intermolecular interaction characteristics, employing methods including the independent gradient model based on Hirshfeld partitioning (IGMH) and energy decomposition analysis based on the force field (EDA-FF).

**Figure 12:**
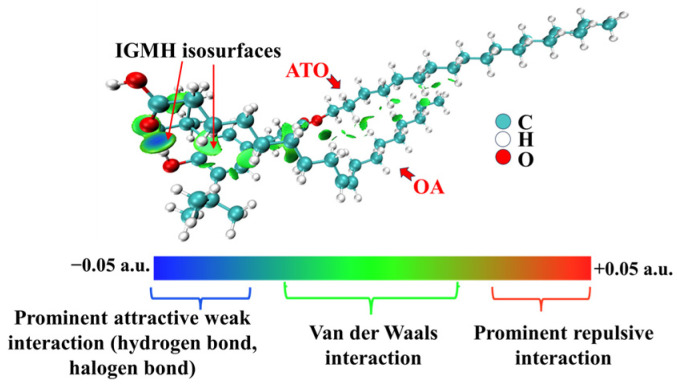
IGMH isosurface map of interaction between OA and ATO.

Specifically, the IGMH method identifies intermolecular interaction regions through isosurfaces of the three-dimensional function δg^inter^, while color projections of sign(λ_2_)ρ distinguish the types and strengths of these interactions [61]. Here, δg^inter^ reflects the variation in electron density gradients between interacting atomic pairs, λ_2_ corresponds to the second eigenvalue of the electron density Hessian matrix, and ρ represents the electron density of the system. In the IGMH analysis, a grid spacing of 0.15 Å was used, with the isosurface value set to 0.005 kcal·mol^−1^·Å^−3^, and the BGR color scale for sign(λ_2_)ρ ranging from −0.05 a.u. to 0.05 a.u. All molecular structures and interaction regions were visualized using Visual Molecular Dynamics (VMD). Taking the OA-ATO molecular complex as an example (Figure 12), the green isosurfaces represent van der Waals interaction regions, while the blue isosurfaces indicate hydrogen-bonding interactions.

**Figure 13:**
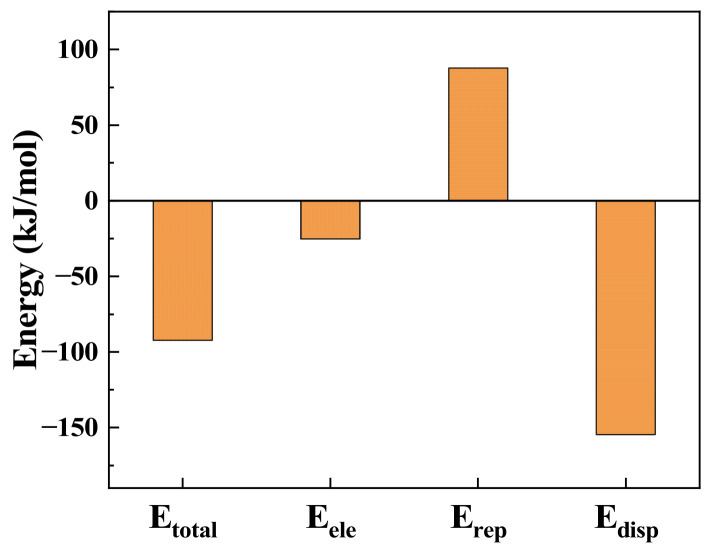
Energy decomposition of interactions between OA and ATO.

The EDA-FF method [62] was employed to calculate the total intermolecular interaction energy and decompose it into physically meaningful components, including electrostatic interaction, Pauli repulsion, and dispersion interaction, thereby enabling a deeper understanding of the nature of intermolecular interactions. Taking the OA-ATO molecular complex as an example (Figure 13), the AMBER force field implemented in *Multiwfn 3.8* yields a total interaction energy (E_total_ = −92 KJ/mol), which is further decomposed into electrostatic (E_ele_ = −25 KJ/mol), Pauli repulsion (E_rep_ = 87 kcal/mol), and dispersion (E_disp_ = −154 KJ/mol) contributions. It should be noted that the EDA-FF results characterize the magnitude and individual contributions of the interaction energy between two fragments, rather than the absolute total energy of the entire molecular complex. Therefore, all energy terms in the EDA-FF analysis are reported in kJ/mol.

## 6. Results and Discussion

### 6.1. WVO-R Formulation Optimization

#### 6.1.1. RSM Analysis Results of WVO-R Formulation Design

**Figure 14:**
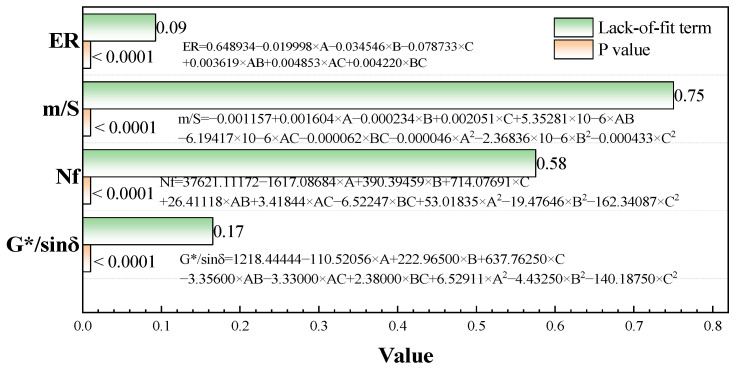
Regression models and ANOVA results for response indicators.

**Figure 15:**
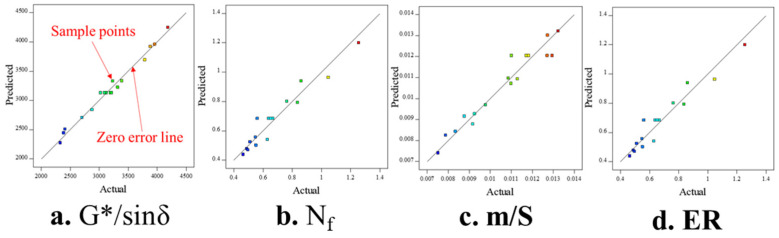
Comparison between the predicted and measured values of response indicators.

The regression models for the four response indicators were developed using *Design-Expert 13*, with the corresponding analysis of variance (ANOVA) results shown in Figure 14. The *p*-values of all models are below 0.0001, indicating high statistical significance, while the lack-of-fit values all exceed 0.05, confirming no significant lack of fit [63]. The adequacy diagnostic results for the four regression models are presented in Table 5. For all models, the difference between the predicted R^2^ and adjusted R^2^ is less than 0.2, and the Adeq Precision values are all greater than 4, indicating that the models provide an adequate signal for navigating the design space [64]. A comparison between the predicted and experimental values of the four response indicators is also shown in Figure 15, where the data points are closely distributed around the zero-error line, demonstrating high fitting accuracy and confirming the suitability of the models for subsequent optimization [65].

**Figure 16:**
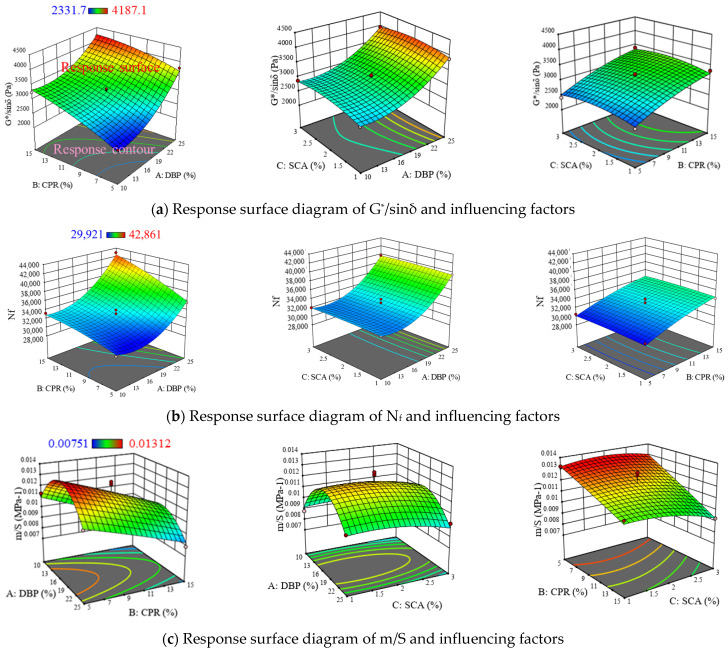

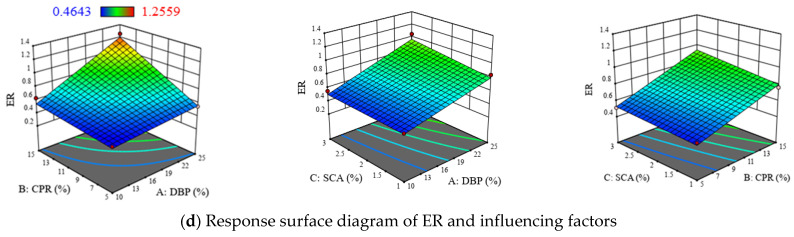
Response surface diagrams of response indicators and influencing factors.

The response surface diagrams illustrating the relationships between the response indicators and influencing factors, as shown in Figure 16, include both three-dimensional response surfaces and contour plots. The response surfaces illustrate the overall trends of the response values as two factors vary, while the degree of contour-line distortion reflects the interaction intensity between two influencing factors [66]. As shown in Figure 16a,b,d, G^*^/sinδ, N_f_, and ER all increase significantly with higher DBP and CPR contents, whereas the effect of SCA content is relatively weak. Strong interactions between DBP and CPR contents are evidenced by the pronounced distortion of the contour lines for G^*^/sinδ and N_f_. For m/S (Figure 16c), the value initially increases and then decreases with increasing DBP content, while exhibiting an overall decreasing trend with CPR content; the influence of SCA content remains relatively minor. Additionally, the contour patterns reveal notable DBP-CPR and DBP-SCA interactions. In summary, variations in DBP and CPR contents are the primary influencing factors and exhibit strong interaction effects across multiple performance indicators, whereas changes in SCA content have a relatively weak impact.

Finally, using the G^*^/sinδ, N_f_, m/S, and ER values of the unaged SK-70# bitumen as target values, the optimized formulation of the WVO-R was determined as WVO:DBP:CPR:SCA:ATO = 100:23.6:14.4:1.7:1. Table 6 presents the predicted and measured values of the four response indicators, with errors below 4%, indicating that the optimized WVO-R formulation obtained via RSM is both accurate and reliable.

#### 6.1.2. Comparison with the Traditional WVO-R Formulation Optimization Framework

**Figure 17:**
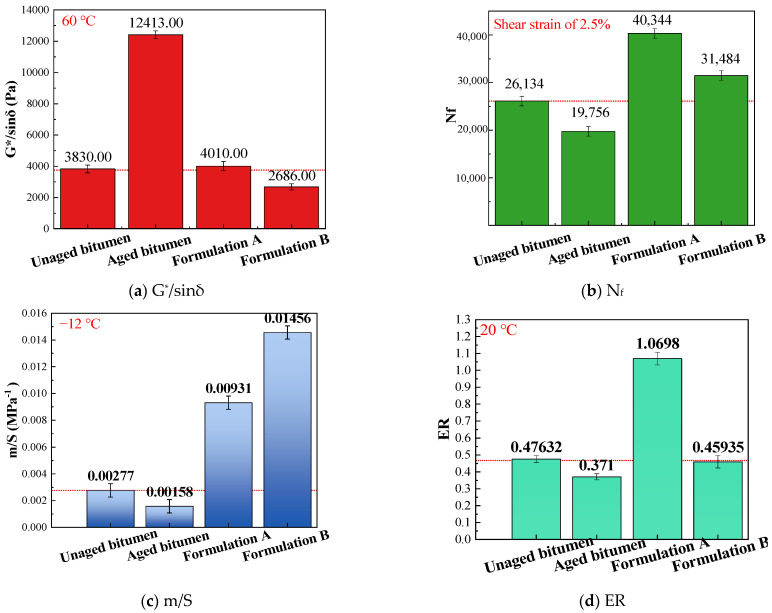
Regeneration effectiveness of different WVO-R optimized formulations.

The penetration, softening point, ductility, and viscosity of the regenerated bitumen were selected as response variables for the RSM analysis, and the corresponding experimental design and results are summarized in Table 7. Using the basic properties of unaged SK-70# bitumen as target values, the optimized WVO-R formulation B was determined as WVO:DBP:CPR:SCA:ATO = 100:10:7.98:2.07:1, while the formulation optimized in the previous section is referred to as formulation A. Figure 17 shows that the regenerated bitumen prepared with formulation B exhibits lower high-temperature resistance to permanent deformation and interfacial water stability than unaged bitumen, and its fatigue life is also significantly inferior to that of formulation A.

These results indicate that the traditional WVO-R formulation optimization method struggles to identify risks associated with high-temperature performance and interfacial water stability. In contrast, an optimization framework dominated by multi-temperature rheological properties and interfacial water stability can effectively avoid such risks and demonstrates superior engineering applicability.

#### 6.1.3. Effect of WVO-R Dosage on Regeneration Effectiveness

**Figure 18:**
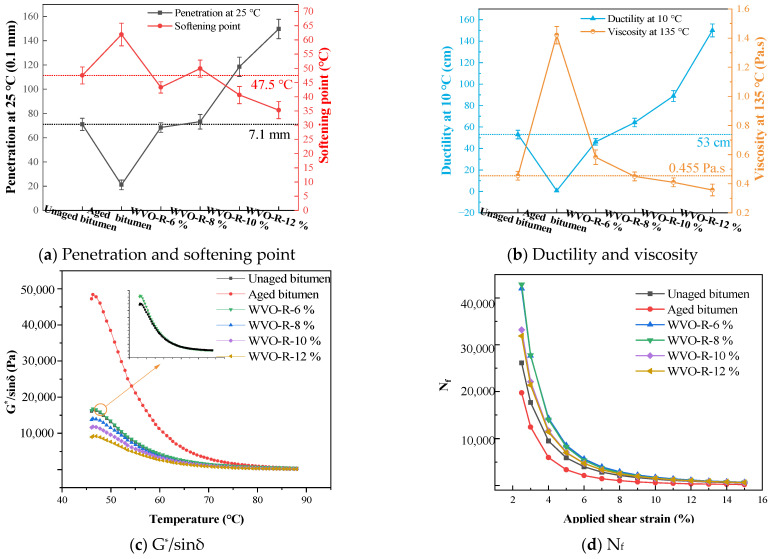

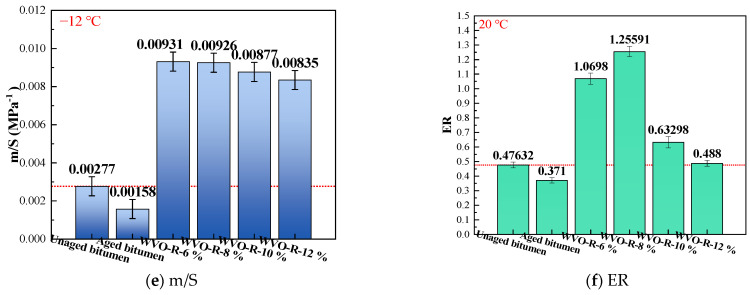
Regeneration effectiveness of different WVO-R dosages.

This section examines the effect of the WVO-R dosage, under the optimal formulation, on regeneration effectiveness. Regenerated bitumen samples were prepared with the WVO-R dosages ranging from 6% to 12%, and their fundamental physical properties, mechanical performance at high, medium, and low temperatures, as well as interfacial water stability, were evaluated. The corresponding results are presented in Figure 18a–f. At the WVO-R dosages of 6–8%, the basic physical properties of regenerated bitumen most closely match those of the unaged bitumen. Analysis of G^*^/sinδ indicates that a 6% WVO-R dosage restores high-temperature resistance to permanent deformation to levels comparable with unaged bitumen. The N_f_ and m/S results indicate that all regenerated bitumen samples exhibit superior medium-temperature fatigue resistance and low-temperature crack resistance compared to the unaged bitumen. Moreover, ER results demonstrate that a 6–8% WVO-R dosage significantly improves the water stability at the regenerated bitumen-aggregate interface. Overall, the optimal WVO-R dosage is determined to be 6–8%.

**Figure 19:**
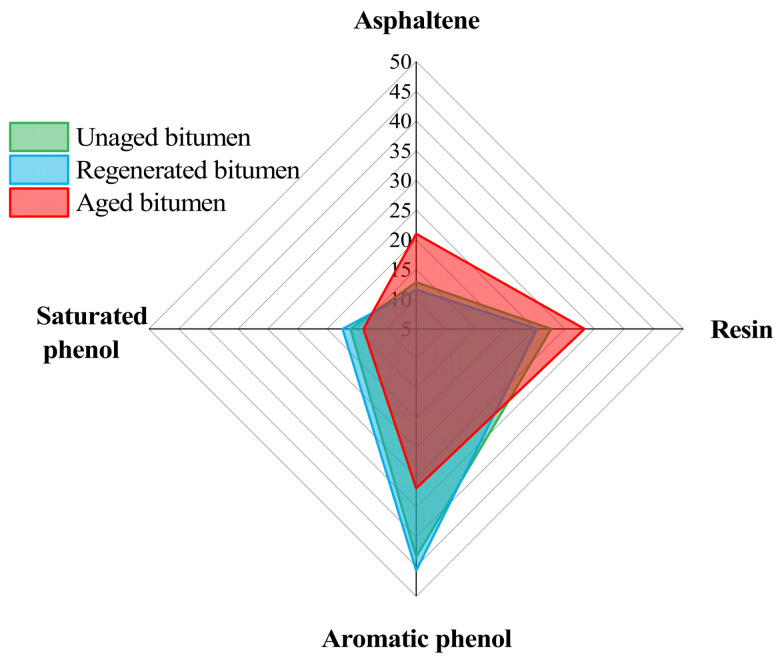
Statistical results of asphalt four-component ratio.

**Figure 20:**
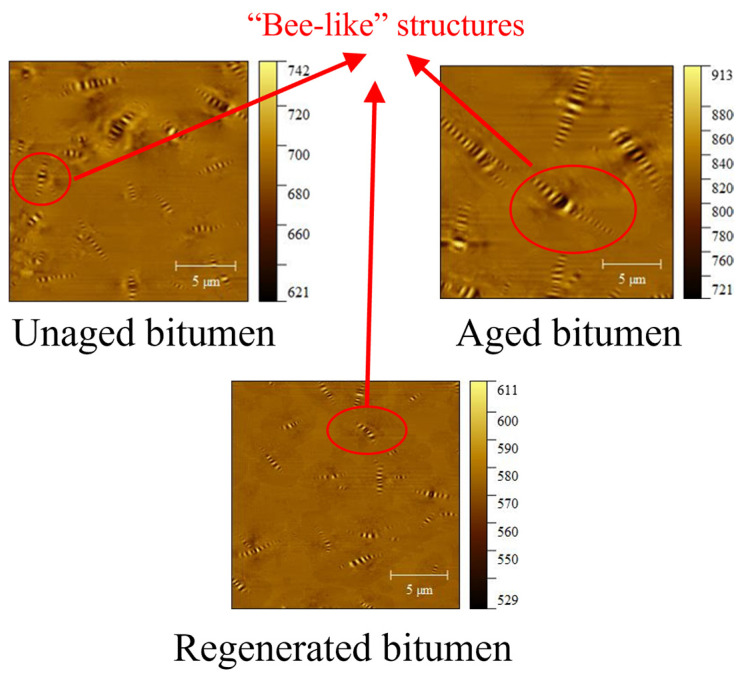
Microscopic morphology of different bitumen.

To further elucidate the regeneration effect, TLC-FID (Thin layer chromatography-flame ionization detection) [67] and AFM (Atomic force microscopy) [68] analyses were performed on the regenerated bitumen (the WVO-R dosage of 7%). As shown in Figure 19, the WVO-R effectively restores the component composition of aged bitumen to a level comparable to that of unaged bitumen. In addition, the number and size of the “bee-like” structures in regenerated bitumen (Figure 20) also return to the unaged state, indicating that the WVO-R can effectively reconstruct the colloidal structure of aged bitumen.

#### 6.1.4. Thermal Behavior Analysis of WVO-R

**Figure 21:**
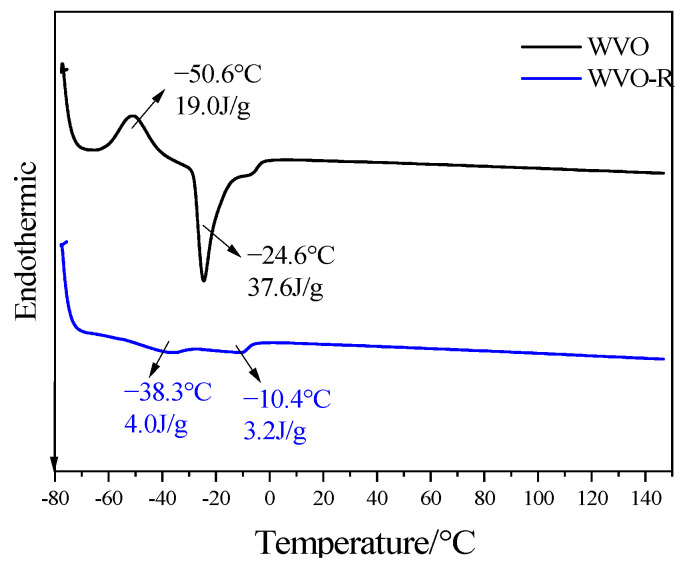
DSC test results of WVO-R and WVO.

DSC analyses were performed on the WVO and WVO-R, and the corresponding heating curves are shown in Figure 21, with the exothermic/endothermic peaks and associated enthalpy values indicated. The WVO heating curve exhibits two characteristic peaks: an exothermic peak at −50.6 °C (ΔH = 19.0 J/g), attributed to cold crystallization, and an endothermic peak at −24.6 °C (ΔH = 37.6 J/g), where the relatively high enthalpy indicates a high degree of crystallinity. In contrast, the WVO-R shows two distinct endothermic peaks at −38.3 °C (ΔH = 4.0 J/g) and −10.4 °C (ΔH = 3.2 J/g), corresponding to the melting of a low-melting-point eutectic and a high-melting-point phase formed through interactions between WVO and the functional components, respectively.

**Figure 22:**
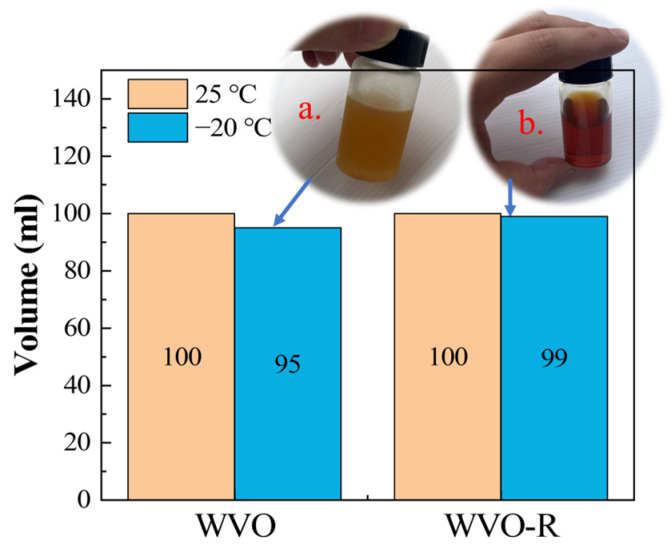
Appearance and volume changes in WVO and WVO-R at low temperature. (**a**) is the appearance of WVO-R at −20 °C; (**b**) is the appearance of WVO at −20 °C.

Notably, the DSC heating curve of WVO-R maintains a stable baseline over the temperature range of 20–150 °C, indicating the absence of significant phase transitions and thus stable thermal behavior. Compared with WVO, the markedly reduced enthalpy of WVO-R suggests a lower degree of crystallinity, which suppresses crystal growth and sedimentation, thereby enhancing storage stability. Furthermore, the disappearance of the cold-crystallization exothermic peak indicates altered thermal behavior, as the functional components promote the uniform dispersion of WVO and inhibit reheating-induced recrystallization [69]. To further support this interpretation, WVO and WVO-R were stored at −20 °C for 3 h. WVO became turbid, lost its flowability, and exhibited noticeable volume shrinkage (Figure 22a), whereas WVO-R remained transparent and flowable, with no obvious macroscopic volume change (Figure 22b). These observations suggest that WVO-R has better low-temperature stability and a lower tendency toward crystallization or phase separation. Overall, these results indicate that WVO-R possesses good mixing uniformity, together with improved thermal and storage stability.

**Figure 23:**
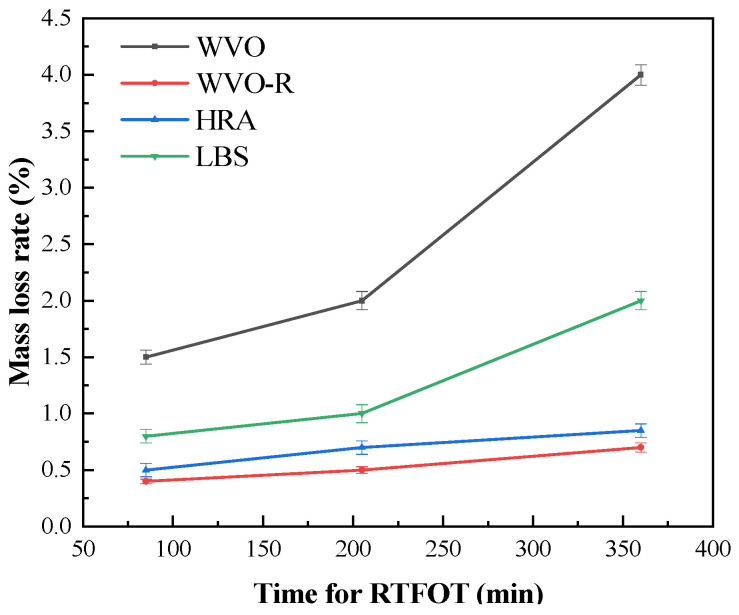
Test results of RTFOT.

In addition, RTFOT was employed to evaluate the anti-aging performance of WVO-R, WVO, and two commercial regenerants (LBS and HRA), with the results shown in Figure 23. The WVO-R exhibits the lowest mass loss among all samples, confirming its superior resistance to thermal aging and the effectiveness of the 1% ATO dosage in its formulation.

### 6.2. Analysis of Compounding Mechanism for WVO-R

#### 6.2.1. Electronic Structure Characteristics Analysis of WVO-R Components

This section systematically describes the differences in electronic structural characteristics among the WVO-R components, with a focus on molecular polarity and electronic reactivity. Molecular polarity is quantified at different dimensions using the dipole moment and the molecular polarity index (MPI), where the dipole moment reflects the overall charge separation within a molecule [70], and MPI captures the spatial fluctuations of the molecular surface electrostatic potential [71]. Molecular electronic reactivity is assessed via the energies of the highest occupied molecular orbital (HOMO), the lowest unoccupied molecular orbital (LUMO), and the corresponding energy gap (ΔE). The calculation formula for ΔE is presented in Equation (5).

(5) $$\Delta E=E_{LUMO}-E_{HOMO}$$

**Figure 24:**
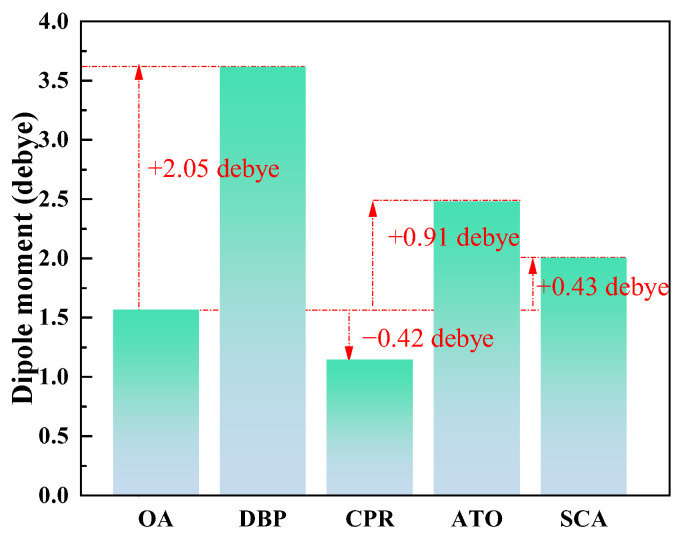
Dipole moments of WVO-R component molecules.

**Figure 25:**
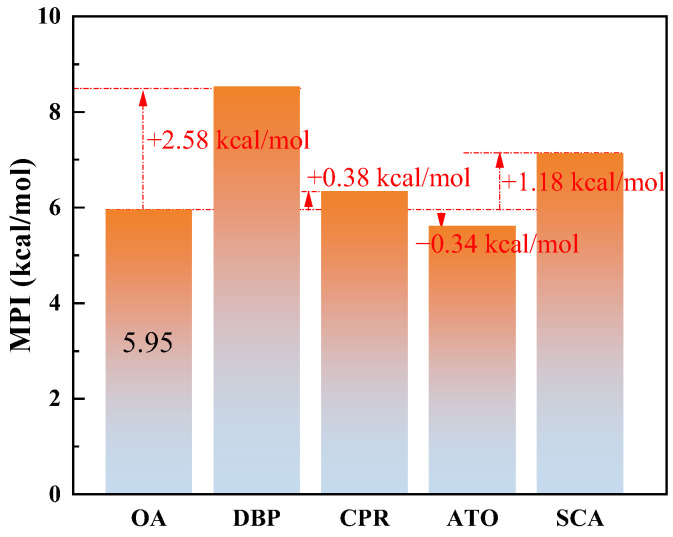
MPI values of WVO-R component molecules.

The dipole moments and MPI values of the WVO-R components are summarized in Figure 24 and Figure 25. CPR, ATO, and SCA exhibit molecular polarity characteristics comparable to those of OA, as evidenced by dipole moment differences in less than 0.91 D and MPI differences in less than 1.18 kcal/mol. In contrast, DBP shows the most pronounced polarity deviation from OA, with differences of 2.05 D in dipole moment and 2.58 kcal/mol in MPI. Overall, except for DBP, the other functional components exhibit good polarity compatibility with OA. The notable polarity difference between DBP and OA highlights the necessity of incorporating SCA in the WVO-R formulation, as its bipolar structure [37] can effectively regulate the polarity mismatch between DBP and OA.

**Figure 26:**
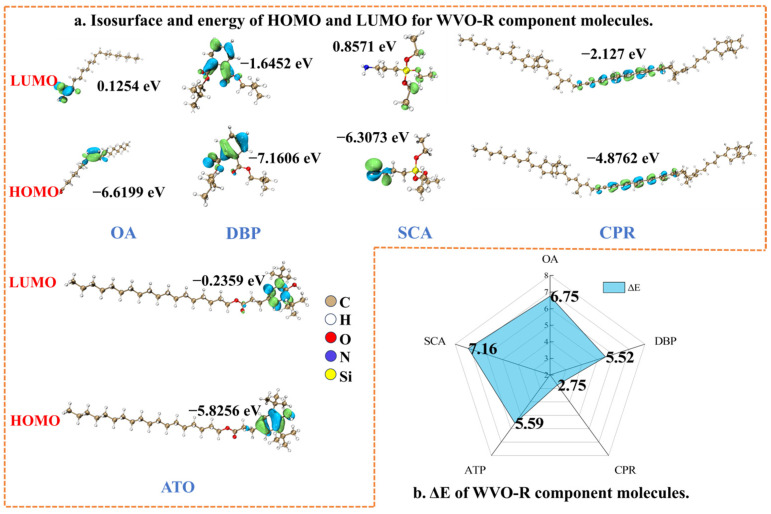
Electronic reactivity analysis of WVO-R component molecules.

In this study, molecular orbitals were visualized via isosurfaces, with light green for the positive phase and light blue for the negative phase. The HOMO and LUMO distributions and energy levels of the WVO-R component molecules are shown in Figure 26a, with their energy gaps (ΔE) shown in Figure 26b. Generally, ΔE < 2 eV indicates high electronic reactivity, while ΔE ≥ 4–5 eV reflects low reactivity and high molecular stability [72]. Here, CPR has a ΔE slightly above 2.5 eV, whereas DBP, OA, SCA, and ATO exceed 5.5 eV, demonstrating that the WVO-R components possess stable electronic structures and low reactivity.

#### 6.2.2. Interaction Analysis Among WVO-R Components

**Figure 27:**
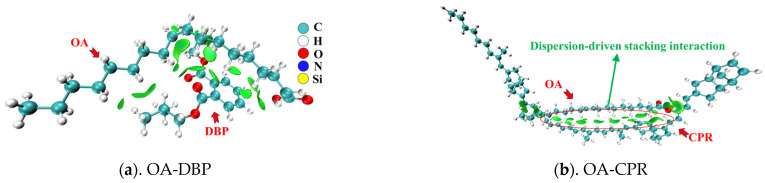

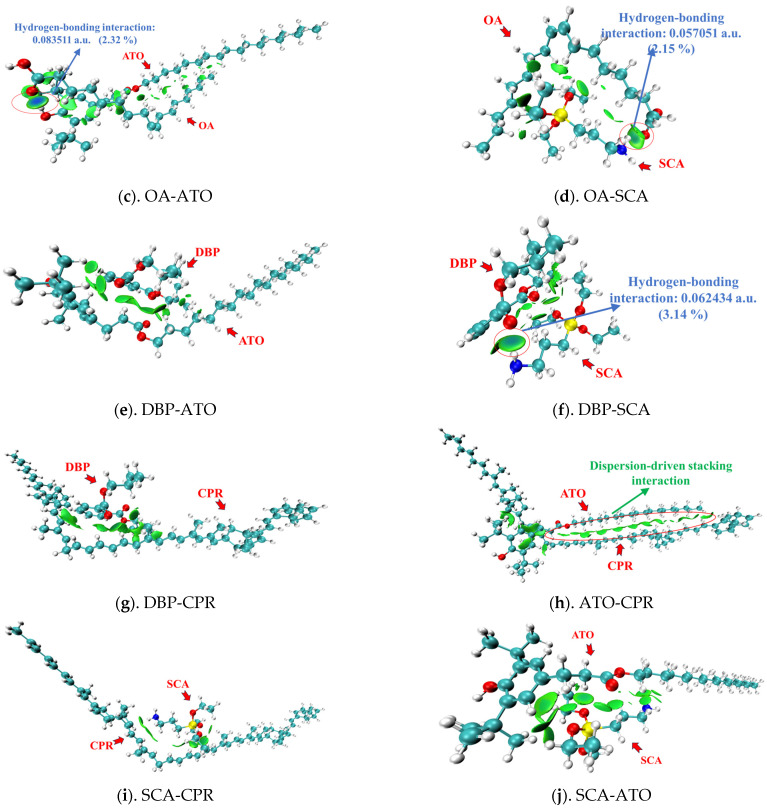
IGMH isosurface maps of interactions among WVO-R component molecules.

The distribution of IGMH isosurfaces among the WVO-R component molecules is shown in Figure 27. Overall, the system is dominated by discontinuous green isosurfaces with localized blue regions, indicating that intermolecular interactions are mainly governed by dispersion force-driven van der Waals interactions, supplemented by localized hydrogen-bonding interactions. Three hydrogen-bonding interactions are identified in the WVO-R system. As shown in Figure 27c,d, two involve the OA carboxyl group interacting with either the ATO hydroxyl group or the SCA amino group, with electron density gradient values and interaction contributions of 0.084 a.u. (2.32%) and 0.057 a.u. (2.15%), respectively. The third hydrogen bond forms between the ester group of DBP and the amino group of SCA, highlighting the polarity-regulating role of SCA, with a value of 0.062 a.u. (3.14%), as shown in Figure 27f. Notably, as shown in Figure 27b,h, multiple sets of parallel green isosurfaces are distributed among OA, the long alkyl chain of ATO, and the conjugated backbone of CPR, indicating pronounced dispersion-driven stacking interactions [73] among these components.

**Figure 28:**
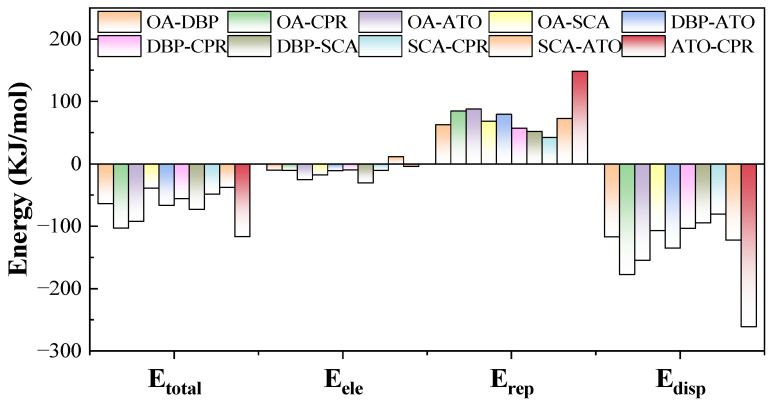
Energy decomposition of interactions among WVO-R component molecules.

The energy decomposition results for the interactions among the WVO-R components are presented in Figure 28. The total interaction energy, dispersion energy, and electrostatic energy are all negative, indicating attractive interactions, whereas the Pauli repulsion term is positive, reflecting its repulsive nature. Dispersion attractions play a decisive role in the binding of the WVO-R components, accounting for the largest proportion of the decomposed energy terms. In the OA-ATO, OA-SCA, and DBP-SCA systems, electrostatic interactions are comparatively more pronounced, with energies of 25, 18, and 30 kJ·mol^−1^, respectively. The strongest dispersion attractions are observed in the OA-CPR and CPR-ATO systems, with values of 177 and 261 kJ·mol^−1^, respectively. Overall, the energy decomposition results are consistent with the IGMH analysis, further confirming the interaction mechanisms among the WVO-R components.

**Figure 29:**
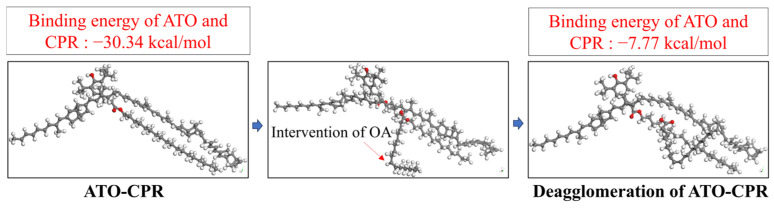
Depolymerization of ATO-CPR.

Notably, the strong dispersion stacking between ATO and CPR may promote the formation of functional component aggregates. Further analysis indicates that the presence of OA significantly weakens their interaction, decreasing the binding energy from 30.34 to 7.77 kcal·mol^−1^, as illustrated in Figure 29. Moreover, given that the ATO dosage is limited to only 1%, the formation of stable CPR-ATO aggregates in the WVO environment is unlikely.

#### 6.2.3. Discussion on Compounding Mechanism of WVO-R

**Figure 30:**
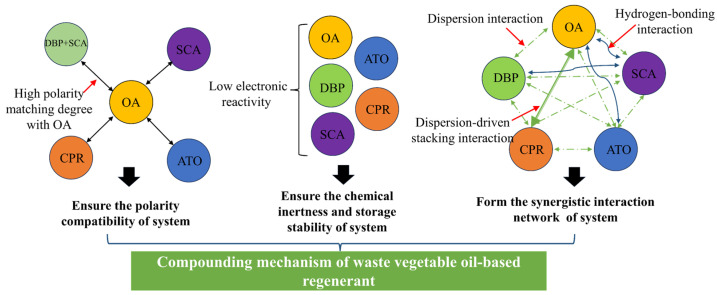
Compounding mechanism diagram of WVO-R.

The superior compounding performance of the WVO-R is attributed to the highly matched electronic structure characteristics of its components and the synergistic formation of a multilevel molecular interaction network. In terms of polarity compatibility, CPR, ATO, and SCA exhibit dipole moments and MPI values close to those of OA, while the polarity disparity between DBP and OA is effectively mitigated by SCA, thereby ensuring overall polarity matching within the system. Regarding molecular electronic reactivity, all components display relatively large HOMO-LUMO energy gaps (ΔE), indicating low chemical reactivity and high electronic stability, which underpins the thermal and storage stability of the WVO-R. The IGMH analysis and energy decomposition results further demonstrate that the WVO-R system establishes a synergistic interaction network dominated by dispersion forces, supplemented by localized dispersion-driven stacking and hydrogen-bonding interactions. Dispersion forces, being weak and non-specific [74], provide a thermodynamic basis for the uniform dispersion of each component, while their cumulative effect enhances intermolecular bonding and structural continuity, as clearly evidenced in the OA-CPR system. Moreover, directional hydrogen bonds between OA and ATO/SCA, as well as between DBP and SCA, further improve component compatibility and impose stable constraints at critical interfaces. In summary, the WVO-R constructs a dynamic and robust molecular aggregation structure by combining inherently stable components, leveraging the bipolar characteristics of SCA to regulate critical polarity mismatches, and relying on ubiquitous dispersion forces alongside the synergistic effects of localized dispersion-driven stacking and directional hydrogen bonding interactions. This structure not only thermodynamically facilitates uniform mixing but also kinetically restrains excessive molecular migration and aggregation, thereby simultaneously achieving high thermal stability, long-term storage stability, and excellent uniformity. The multi-component compounding mechanism of the WVO-R is illustrated in Figure 30.

### 6.3. Analysis of Regeneration Mechanism for WVO-R

#### 6.3.1. Interaction Analysis Between WVO-R Components and Asphaltene

**Figure 31:**
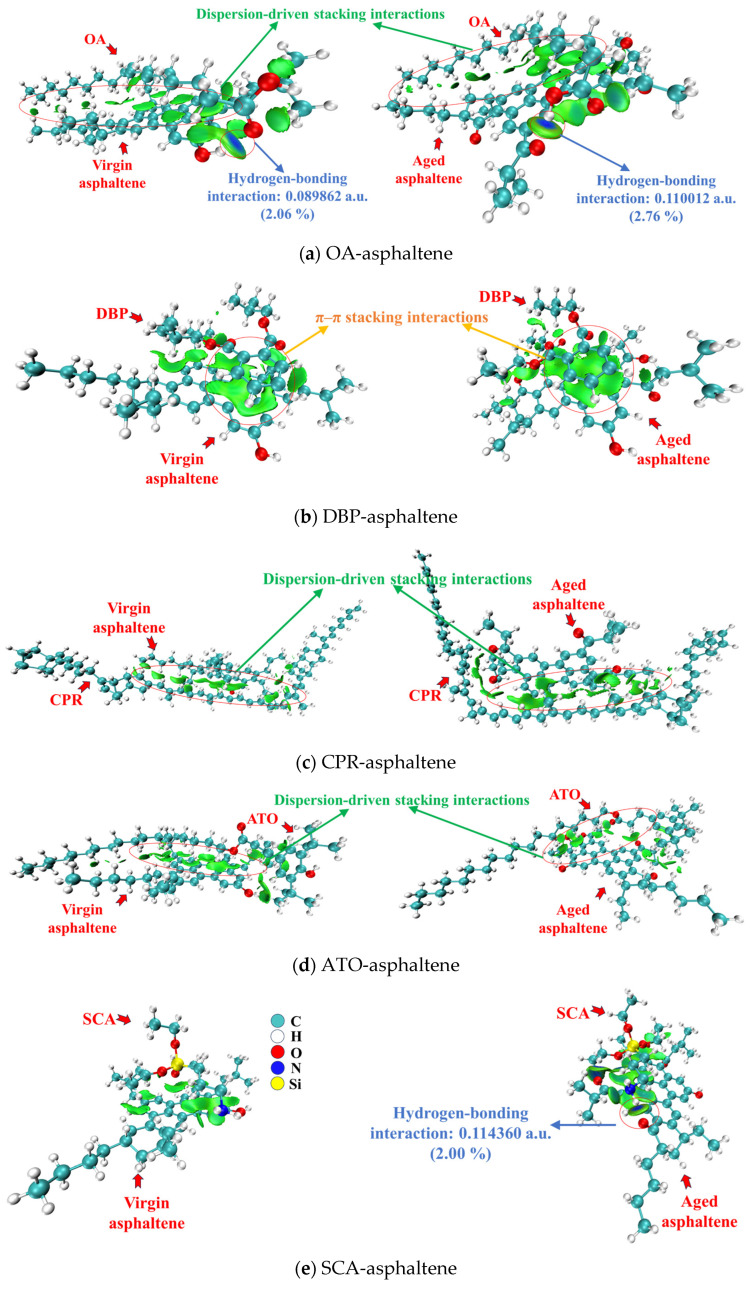
IGMH isosurface maps of interactions between WVO-R components and asphaltene.

The IGMH isosurface maps illustrate the noncovalent interaction characteristics between the WVO-R components and both virgin asphaltene (VAS) and aged asphaltene (AAS), as shown in Figure 31. In Figure 31a, multiple sets of parallel green isosurfaces appear between the alkyl chain of OA and the aromatic regions of asphaltenes, indicating pronounced dispersion-driven stacking interactions. Simultaneously, the carboxyl group of OA forms hydrogen bonds with the hydroxyl group of VAS and the carbonyl group of AAS, with corresponding electron density gradient values and interaction contributions of 0.084 a.u. (2.32%) and 0.057 a.u. (2.15%), respectively. In Figure 31b, relatively continuous and extensive green isosurfaces are observed between the benzene ring of DBP and the aromatic regions of asphaltenes, exhibiting typical π–π stacking characteristics [75]. Figure 31c,d show that both the conjugated backbone of CPR and the long alkyl chain of ATO engage in pronounced dispersion-driven stacking interactions with the aromatic regions of asphaltenes. In Figure 31e, relative to VAS, the amino group of SCA forms a pronounced hydrogen bond with the carbonyl group of AAS, exhibiting an electron density gradient of 0.084 a.u. (2.32%). Overall, the WVO-R components predominantly participate in dispersion-driven stacking interactions with the aromatic regions of asphaltenes, while OA and SCA preferentially form hydrogen bonds with the polar functional groups generated during aging.

**Figure 32:**
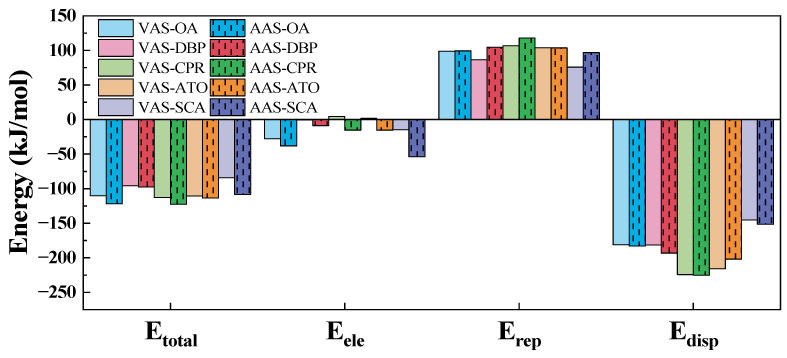
Energy decomposition of interactions between WVO-R components and asphaltene.

The energy decomposition results indicate that all WVO-R components exhibit strong binding with asphaltenes, with total interaction energies generally approaching or exceeding 100 kJ/mol (Figure 32). Dispersion interactions dominate the decomposed energy contributions, remaining at relatively high levels of 145–225 kJ/mol. Notably, following asphaltene aging, its electrostatic interactions with the WVO-R components are generally enhanced, with the most pronounced increase observed for SCA, from 14.92 kJ/mol to 53.95 kJ/mol. This enhancement accounts for the stronger binding between AAS and the WVO-R components. Overall, the energy decomposition results further confirm the interaction mechanisms between the WVO-R components and asphaltenes.

#### 6.3.2. Electronic Structure Characteristics Analysis of Asphaltene Molecular Complexes

**Figure 33:**
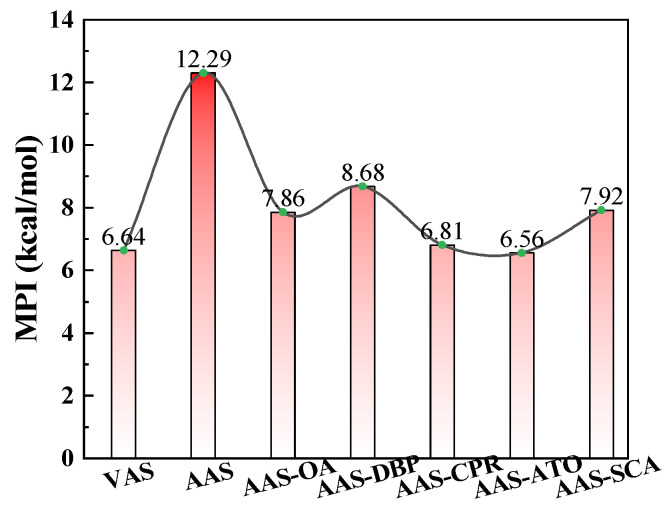
MPI of AAS and its molecular complexes.

**Figure 34:**
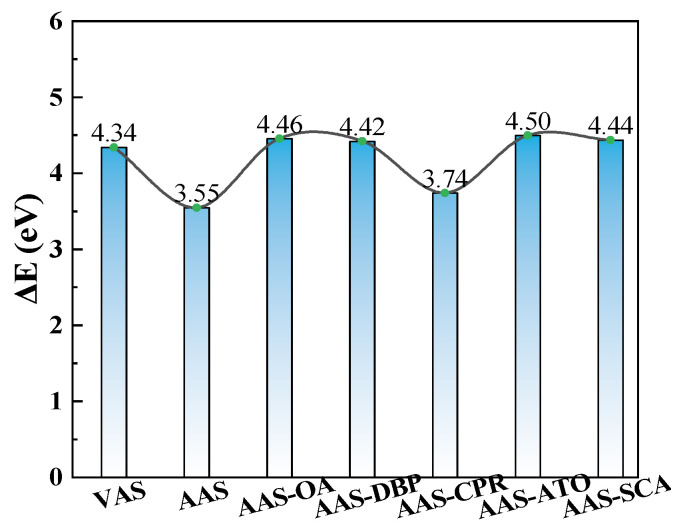
E of AAS and its molecular complexes.

This section further examines the differences in electronic structure characteristics of the molecular complexes formed between AAS and the WVO-R components. The surface electrostatic potential gradient and electronic reactivity of these molecular complexes were quantified using MPI and ΔE, with the statistical results presented in Figure 33 and Figure 34.

The MPI statistical results for AAS and its molecular complexes are presented in Figure 33. The MPI values of all molecular complexes are markedly lower than those of AAS and approach the level of VAS. Among these, CPR and ATO achieve the greatest MPI reduction primarily through the shielding of polar sites by their nonpolar backbones, whereas OA and SCA mainly weaken local surface electrostatic potential gradients via hydrogen-bonding interactions. In contrast, DBP exhibits a relatively limited MPI reduction due to its higher intrinsic polarity. Meanwhile, the ΔE values of all molecular complexes are higher than that of AAS (Figure 34), with most stabilizing at approximately 4.4 eV and approaching the level of VAS. Overall, the incorporation of the WVO-R components with AAS synergistically modulates the surface electrostatic potential gradient and electronic reactivity of AAS, restoring its electronic structure characteristics to levels comparable with those of VAS.

#### 6.3.3. Discussion on AAS Regeneration Mechanism of WVO-R

**Figure 35:**
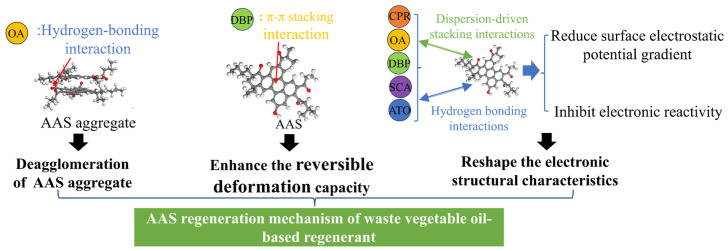
AAS regeneration mechanism of WVO-R.

The WVO-R achieves molecular-level regeneration of AAS through the synergistic action of its multiple components. Regarding the self-aggregation characteristics of AAS [76], the carboxyl group of OA forms directional hydrogen bonds with oxidized polar sites in AAS. This interaction weakens the polar associations within AAS aggregates, thereby inducing their deagglomeration, which is consistent with previous studies [10,27]. Morphological evidence for this deagglomeration is provided by the restoration of the “bee-like” structures in regenerated bitumen. DBP adsorbs onto the aromatic region of AAS via π–π stacking, acting as a flexible buffer layer that helps enhance the reversible deformation capacity of AAS [77], as evidenced by the superior fatigue resistance and low-temperature flexibility of the regenerated bitumen. The overall components of WVO-R establish stable molecular contacts through dispersion-driven stacking interactions and precisely modulate AAS polarity defects via directional hydrogen bonds. This synergistic action smooths the surface electrostatic potential gradient and suppresses the electronic reactivity of AAS, thereby reshaping its electronic structural characteristics to levels comparable with those of VAS. The AAS regeneration mechanism of the WVO-R is shown in Figure 35.

## 7. Extended Discussion of Proposed Work

Although the present work systematically investigates the formulation optimization framework, regeneration effectiveness, thermal characteristics, and molecular interaction mechanisms of WVO-R, the applicability of these findings to asphalt mixture behavior still requires a cautious and well-defined discussion. In light of the binder-scale results obtained in this study and the current state of the art in regenerated bitumen materials, the optimized WVO-R can be reasonably expected to facilitate the performance recovery of recycled asphalt mixtures, especially in terms of improved workability, enhanced resistance to intermediate- and low-temperature cracking, and potentially better moisture stability under properly controlled dosage conditions. Future work will focus on comprehensive validation using recycled asphalt mixtures, including rutting, cracking, moisture damage, and long-term durability tests.

It should be noted, however, that OA alone cannot fully represent the compositional complexity of WVO, nor can a single asphaltene molecule capture the structural diversity of real asphaltenes. Therefore, the present simulations are mainly intended to reveal the dominant interaction mechanisms and their evolution trends in the system. Future work will focus on constructing a more realistic multi-component WVO model, including saturated and unsaturated fatty acids as well as heteroatom-containing species, and introducing asphaltene molecules with diverse structural motifs to better reflect the complexity of actual materials.

## 8. Conclusions

This study optimizes the WVO-R formulation using RSM, evaluates its regeneration efficiency, optimal dosage, and thermal behavior through multi-scale performance tests, and elucidates the multi-component compounding and regeneration mechanisms via systematic analyses of electronic structure characteristics and intermolecular interactions. The main conclusions are as follows:

(1) RSM analysis shows that the contents of DBP and CPR significantly affect the high-, intermediate-, and low-temperature performance as well as the interfacial water stability of the regenerated bitumen, with a strong interaction observed between the two. The optimized WVO-R formulation is WVO:DBP:CPR:SCA:ATO = 100:23.6:14.4:1.7:1.
(2) The traditional WVO-R formulation optimization method struggles to identify risks related to high-temperature performance and interfacial water stability, whereas an optimization framework dominated by multi-temperature rheological properties and interfacial water stability effectively mitigates these risks and demonstrates superior engineering applicability.
(3) The optimal comprehensive performance of regenerated bitumen is achieved at a WVO-R dosage of 6–8% under the optimized formulation. Microscopic analyses further indicate that this dosage effectively restores the four-component balance of aged bitumen and reconstructs its colloidal structure.
(4) DSC analysis indicates that WVO-R exhibits low crystallinity, a stable high-temperature heating curve, and modified thermal behavior, confirming its excellent thermal and storage stability as well as good mixing homogeneity. Furthermore, RTFOT results demonstrate its outstanding aging resistance.
(5) The WVO-R forms a dynamic and stable molecular aggregate structure by integrating inherently stable components, leveraging the bipolar characteristics of SCA to regulate critical polarity mismatches of DBP, and relying on a synergistic interaction network dominated by dispersion forces, supplemented by localized stacking and hydrogen-bonding interactions.
(6) The WVO-R achieves molecular-level regeneration of AAS through the synergistic action of its multiple components: OA depolymerizes AAS aggregates via hydrogen bonding; DBP enhances its reversible deformation capability through π-π stacking; and the synergistic effects of dispersion-driven stacking and directional hydrogen bonding induced by the WVO-R smooth the surface electrostatic potential gradient and suppresses electronic reactivity of AAS, thereby restoring its electronic structural characteristics to levels comparable with those of VAS.

In summary, the proposed multi-objective formulation optimization strategy, together with the clarified multi-component compounding and regeneration mechanisms, provides a solid theoretical foundation and practical engineering reference for the precise design and performance regulation of WVO-Rs. The main innovations of this work are as follows: (i) proposing a multi-objective optimization strategy dominated by multi-temperature rheological properties and interfacial water stability, overcoming the limitations of traditional methods that rely solely on basic bitumen indices; (ii) systematically revealing, for the first time, the multi-component compounding mechanisms of WVO-R from the perspectives of electronic structure characteristics and intermolecular interactions; and (iii) elucidating the molecular-level regeneration mechanisms of aged bitumen through the synergistic actions of WVO-R components.

## Figures and Tables

**Table 1:** Composition analysis result of WVO.

Number	Compound Name	Molecular Formula	Peak Area	Content (%)
1	Oleic acid	C_18_H_34_O_2_	286,694,455	38.97
2	5-Tridecyl-1,2-oxathiolane 2,2-dioxide	C_16_H_32_O_3_S	89,885,209	12.22
3	Methyl palmitate	C_17_H_34_O_2_	2,340,819	0.32
4	Palmitic acid	C_16_H_32_O_2_	26,780,839	3.64
5	2-(9-Decen-1-yl)-4,4,5,5-tetramethyl-1,3,2-dioxaborolane	C_16_H_31_BO_2_	28,440,818	3.87
6	Geranyl formate	C_11_H_18_O_2_	33,424,362	4.54
7	(2E)-1-(1-Piperidinyl)-2-decen-1-one	C_15_H_27_NO	40,487,003	5.50
8	Tert-butyl 2,7-diazaspiro [3.5]nonane-7-carboxylate	C_12_H_22_N_2_O_2_	58,794,367	7.99
9	3,5-Ditert-butyl catechol	C_14_H_22_O_2_	5,081,983	0.69
10	Octadecyl Isocyanate	C_19_H_37_NO	10,462,484	1.42
11	N-(2-Hydroxybenzyl)-1-dodecanaminium	C_19_H_34_NO	8,139,821	1.11

**Table 2:** Basic performances of functional components for WVO-R.

Properties	CPR	ATO	SCA	DBP	Unit	Specification
Density	0.962	0.991	0.946	1.044	g/cm^3^	ASTM D4052
Water content	0.082	0.052	0.063	0.050	%	ASTM E203
Viscosity (60 °C)	—	—	0.002	0.005	Pa·s	ASTM D445
Softening/melting point	115	52	—	—	°C	ASTM E28/D7346

**Table 3:** Basic technical indicators of unaged and aged bitumen.

Material Type	Penetration at 25 °C	Softening Point	Ductility at 10 °C	Viscosity at 135 °C
Unaged bitumen	7.1 mm	47.5 °C	53 cm	0.455 Pa·s
Aged bitumen	2.11 mm	61.9 °C	0.6 cm	1.42 Pa·s

**Table 4:** Experimental combinations and corresponding results for WVO-R formulation design.

Number	Factor A	Factor B	Factor A	Response 1	Response 2	Response 3	Response 4
	DBP, %	CPR, %	SCA, %	G^*^/sinδ, Pa	N_f_	m/S, Mpa^−1^	ER
1	10	10	1	2704	31,867.1	0.0087	0.4883
2	10	5	2	2331.7	29,921.2	0.0113	0.4643
3	10	15	2	3235.1	33,196.9	0.0075	0.6294
4	10	10	3	2878.1	31,981.3	0.0083	0.5524
5	17.5	5	1	2389.1	30,315.7	0.0132	0.4972
6	17.5	15	1	3320.5	34,124.5	0.0109	0.7631
7	17.5	10	2	3024.1	32,902.7	0.0127	0.5612
8	17.5	10	2	3124.2	33,112.6	0.0117	0.6453
9	17.5	10	2	3089.1	33,945.3	0.0118	0.6394
10	17.5	10	2	3213.3	32,017.5	0.0129	0.6533
11	17.5	10	2	3193.3	32,547.9	0.0110	0.6694
12	17.5	5	3	2411.3	30,452.7	0.0127	0.5114
13	17.5	15	3	3390.3	34,131.2	0.0092	0.8617
14	25	10	1	3883.6	39,417.7	0.0097	0.8372
15	25	5	2	3787.1	35,623.4	0.0108	0.5479
16	25	15	2	4187.1	42,861.1	0.0078	1.2559
17	25	10	3	3957.8	39,634.5	0.0091	1.0472

**Table 5:** Adequacy diagnosis results of four regression models.

	R^2^	Adjusted R^2^	Predicted R^2^	Adeq Precision
G^*^/sinδ	0.9844	0.9642	0.8209	24.6994
N_f_	0.9842	0.9639	0.8931	24.2244
m/S	0.9409	0.8650	0.7043	11.1107
ER	0.9292	0.8868	0.7446	16.4938

**Table 6:** Verification of optimal formulation.

Project	G^*^/sinδ (Pa)	N_f_	m/S (Mpa^−1^)	ER
Measured data	4130.6	42,008.46014	0.009312	1.0698
Forecast data	4010.0351	40,344.3628	0.009383	1.0746
Error (%)	2.9%	3.96%	0.7624%	0.4486%

**Table 7:** Experimental combinations and corresponding results.

Number	Factor A	Factor B	Factor A	Response 1	Response 2	Response 3	Response 4
	DBP, %	CPR, %	SCA, %	Penetration at 25 °C, 0.1 mm	Softening Point, °C	Ductility at 10 °C, cm	Viscosity at 135 °C, Pa·s
1	10	5	2	73.6	46.3	15.4	0.463
2	10	10	3	72.3	47.4	15.1	0.456
3	10	10	1	68.9	48.8	13.8	0.471
4	10	15	2	61	53.6	11.5	0.488
5	17.5	5	1	72	48.3	17.6	0.442
6	17.5	10	2	69.3	48.5	16.3	0.448
7	17.5	10	2	69.3	48.5	16.3	0.448
8	17.5	15	1	66.9	50.2	14.3	0.481
9	17.5	10	2	69.3	48.5	16.3	0.448
10	17.5	10	2	69.3	48.5	16.3	0.448
11	17.5	15	3	65.1	49.1	13.6	0.469
12	17.5	10	2	69.3	48.5	16.3	0.448
13	17.5	5	3	75.6	46.3	17.8	0.446
14	25	10	1	71.6	49.3	17.7	0.440
15	25	5	2	81.1	43.2	10.5	0.416
16	25	15	2	68.6	48.1	15.8	0.445
17	25	10	3	75.8	47.1	17.8	0.424

## Data Availability

The original contributions presented in this study are included in the article. Further inquiries can be directed to the corresponding author.

## Abbreviations

DFT	Density functional theory
GC-MS	Gas chromatography-mass spectrometry
PAV	Pressure aging vessel
DSR	Dynamic shear rheometer
BBR	Bending beam rheometer
VMD	Visual Molecular Dynamics
OA	Oleic acid
CPR	C5 petroleum resin
IGMH	Independent gradient model based on Hirshfeld partition
EDA-FF	Energy decomposition analysis based on force field
DBP	Dibutyl phthalate
ATO	Antioxidant 1076
SCA	Silane coupling agent
WVO	Waste vegetable oil
WVO-R	Waste vegetable oil–based regenerant
HOMO	Highest occupied molecular orbital
LUMO	Lowest unoccupied molecular orbital
MPI	Molecular polarity index
RTFOT	Rolling thin-film oven test
ΔCI	Absolute change of carbonyl index
FTIR	Fourier transform infrared spectroscopy
BSSE	Basis set superposition error
TLC-FID	Thin layer chromatography-flame ionization detection
AFM	Atomic force microscopy
VAS	Virgin asphaltene
AAS	Aged asphaltene
